# Serum high-density lipoprotein level and prognosis of ovarian cancer

**DOI:** 10.1097/MD.0000000000035561

**Published:** 2023-10-13

**Authors:** Jiang Hongyan, Chen Pengcheng, Zhu Chihong, Qian Xiaoqian, Wan Danying, Feng Jianguo

**Affiliations:** a The Second Affiliated Hospital Zhejiang University School of Medicine, Hangzhou, Zhejiang, China; b Institute of Cancer Research and Basic Medical Sciences of Chinese Academy of Sciences, Cancer Hospital of University of Chinese Academy of Sciences, Zhejiang Cancer Hospital, Hangzhou, Zhejiang, China; c Zhejiang Key Laboratory of Diagnosis and Treatment Technology on Thoracic Oncology (Lung and Esophagus), Hangzhou, Zhejiang, China.

**Keywords:** density lipoprotein (HDL) prognosis, ovarian cancer high

## Abstract

This study aimed to investigate the prognostic value of serum high-density lipoprotein (HDL) level in patients with ovarian cancer. This study enrolled 152 patients diagnosed with ovarian cancer and 119 patients with benign ovarian tumors. The associations of patient characteristics and disease with survival were determined using Cox regression analysis, *t* tests, analysis of variance for multiple-group comparisons, and chi-square tests. The potential association between HDL levels and the clinical characteristics of the disease was also analyzed. The diagnostic value of HDL was estimated using receiver operating characteristic curve analysis and calculation of the area under the curve. Progression-free survival and overall survival were determined using the Kaplan–Meier method, and their associations with patient and pathological variables, including HDL, were determined using the log-rank test. The median serum HDL was 1.15 mm measured in 152 patients with ovarian cancer and 1.30 mm in 119 patients with benign ovarian tumors (*P* = .000054). The receiver operating characteristic curve analysis yielded an area under the curve of 0.735 for serum HDL levels. Serum HDL levels were significantly associated with tumor pathological types (non-serous vs serous, *P* < .05). No association was observed between serum HDL levels and patient age, age at menarche or marriage, number of children, tumor grade, or clinical stage (*P* > .05). Patients with high serum HDL levels had a longer progression-free survival and overall survival than those with low serum HDL levels. Serum HDL levels are an independent prognostic factor for ovarian cancer.

## 1. Introduction

Ovarian cancer is the third most common gynecological malignancy and has the highest mortality rate.^[1,2]^ More than 200,000 new cases^[3]^ are recorded worldwide each year. Because of a lack of early symptoms, 70% to 80% of patients are diagnosed with advanced-stage disease, which currently has a poor prognosis with a low cure rate. A retrospective analysis of European patients reported an average 5-year survival rate of 37%,^[4]^ and the 5-year survival rate of patients with ovarian cancer in China was < 30%. Many potential prognostic markers have been investigated for ovarian cancer, but specific diagnostic indicators are currently unknown. Notably, some ovarian cancer patients experience early recurrence and metastasis within a short period of curative surgery, whereas others diagnosed with advanced ovarian cancer can survive for a long time.^[5]^ Thus, the clinical management of ovarian cancer remains challenging, and more effective prognostic indicators that allow individualized treatment are needed to improve treatment effectiveness and patient survival.

Cholesterol is essential not only for cell membrane structures, but also for the synthesis of hormones and other substances that maintain the physiological functions of cells. However, studies have shown that high serum cholesterol levels increase the risk of malignancy and the development of a variety of tumors, including breast, prostate, colorectal, stomach, and kidney cancer.^[6–10]^ High cholesterol levels also indicate worse prognosis in cancer patients.^[11,12]^ Studies have also revealed an association between blood cholesterol and cancer risk as well.^[13]^

Notably, high-density lipoprotein (HDL) transports phospholipids and regulates cholesterol content by reversing endogenous cholesterol from peripheral tissues to the liver as cholesteryl ester.^[14]^ Thus, it is of interest to determine whether HDL levels could be associated with ovarian cancer risk as a prognostic marker. In this study, we evaluated perioperative HDL levels in ovarian cancer patients and in patients diagnosed with benign ovarian tumors, and conducted analyses to determine whether serum HDL could serve as a diagnostic or prognostic marker for ovarian cancer.

## 2. Material and methods

### 2.1. Ethical compliance

The internal ovarian cancer cohort research protocol was approved by the Clinical Research Ethics Board of the Zhejiang Cancer Hospital (No: IRB-2022-540). All procedures performed in this study involving human participants were in accordance with the ethical standards of the institutional and/or national research committee and the 1964 Helsinki Declaration and its later amendments or comparable ethical standards.

### 2.2. Study population

A total of 152 patients diagnosed with ovarian cancer and 119 with benign ovarian tumors who were treated between June 2008 and June 2015 were enrolled in this study. The diagnosis was pathologically confirmed both preoperatively and postoperatively. Pathohistological diagnosis was based on the 2014 World Health Organization Ovarian Cancer histological classification criteria, which divided the cancer cases into 105 serous and 47 non-serous carcinomas (34 endometrioid carcinomas, 9 myxoid carcinomas, and 4 clear-cell carcinomas). Following the 2010 American Joint Committee on Cancer ovarian and primary peritoneal cancer TNM and FIGO staging criteria, these cancer patients were further diagnosed as stage I (4 cases with 1 stage Ia and 3 stage Ic), stage II (14 cases with 1 stage IIa, 4 stage IIb, and 9 stage IIc), stage III (123 cases with 14 stage IIIb and 109 stage IIIc), and stage IVa (11 cases). The benign tumors included 62 ovarian cysts, 44 serous cysts, and 13 mucinous cystadenomas. The mean age of the cancer patients was 54.5 ± 9.0 years, and the median age was 54 (range 37–78) years. None of the patients had received preoperative radiotherapy, chemotherapy, or other treatments.

### 2.3. HDL assay, patient follow-up and statistical analysis

Serum HDL levels were determined using a Hitachi 7600 autoanalyzer. Reagents for HDL assay and quality control were purchased from Japan’s First Chemical Co., Ltd.

Patients were followed-up by personal telephone calls or calls to their family members until October 30, 2016. The longest follow-up period was 86 months and the median follow-up period was 54 months. Five patients (3.3%) lost contact during the follow-up period. Receiver operating characteristic (ROC) curve analysis of benign ovarian tumors was used to determine the cutoff value for serum HDL stratification. Correlations between the clinical characteristics and serum HDL levels were determined using *t*-test. Analysis of variance was used to compare differences among multiple groups. Disease stage data were compared using the chi-square test. Differences in survival and disease progression associated with serum HDL levels were analyzed using the Kaplan–Meier method and log-rank test. A multifactorial analysis of survival was performed using Cox regression analysis. Statistical significance was set at *P* < .05.

### 2.4. Data access statement

Research data supporting this publication are available upon request.

## 3. Results

### 3.1. Serum HDL levels measured in ovarian cancer and benign ovarian tumor patients

**Figure 1.**
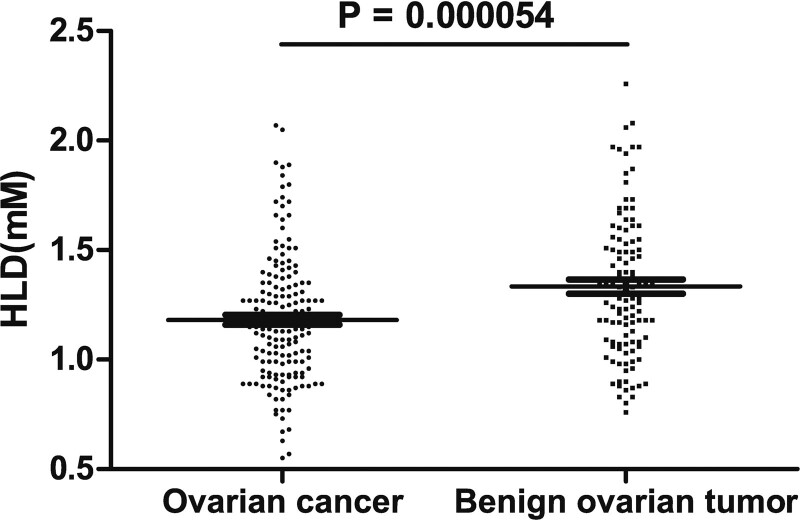
Serum HDL levels in ovarian cancer and benign ovarian tumor patients. Graphics shows the serum HDL concentrations measured in patients diagnosed with ovarian cancer or benign ovarian tumors. *P* < .005 indicates the statistical significance. HDL = high-density lipoprotein.

The serum HDL concentrations range from 0.55 to 2.07 (median level of 1.15) for ovarian cancer patients, and 0.55 to 2.07 (median level of 1.30) for patients diagnosed with benign ovarian tumors, respectively. Statistical analysis showed a significant difference (*P* = .000054) between the 2 patient groups (Fig. 1).

### 3.2. Serum HDL level, cutoff value, and diagnostic value.

**Figure 2.**
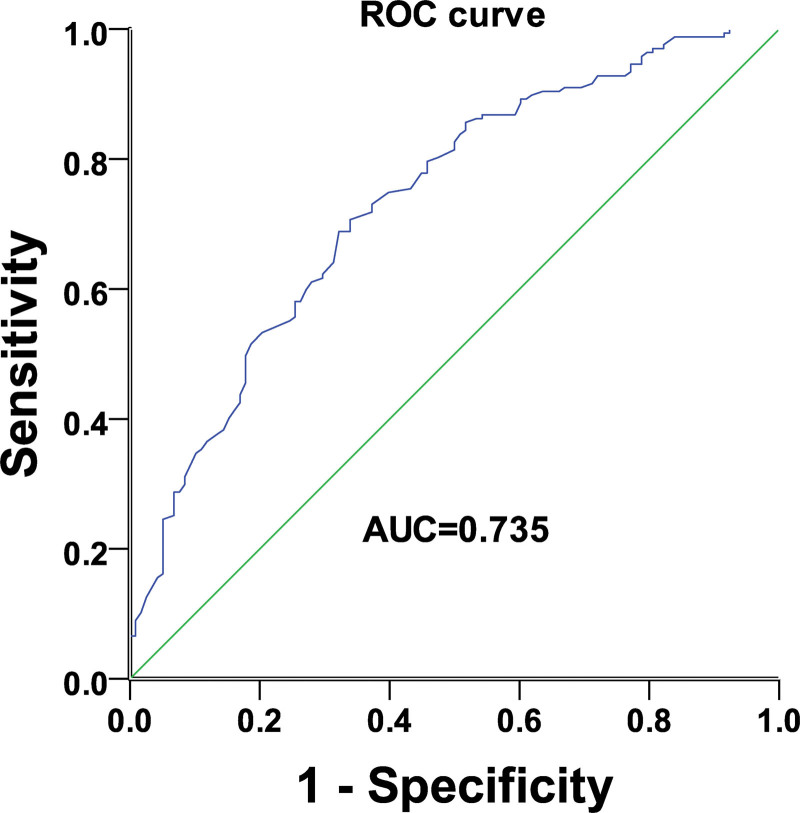
ROC analysis. ROC analysis was used to determine the cutoff value of the serum HDL levels for stratifing the patients with high serum HDL or low serum HDL. HDL = high-density lipoprotein, ROC = receiver operating characteristic.

ROC curve analysis of the ovarian cancer patients indicated that a cutoff value for serum HDL level of 1.2 mm had high specificity and sensitivity (Fig. 2, (area under curve = 0.735), which was thus used to stratify patients into high (n = 88) and low (n = 64) serum HDL level groups. Results from the *t* test, chi-square test, and 1-way Analysis of variance test indicated that there was an association between serum HDL levels and the pathologic types, that is, ovarian cancer versus benign ovarian tumors. The *t* test (*P* = .019) and chi-square test (*P* = .016) showed that marital age, age at menarche, number of children, tumor grade, and tumor stage were not associated with patient pathology (*P* > .05, Table 1).

**Table 1:** Correlations of serum HDL level with clinicapathological factors of ovarian cancer patients.

Factors	Patients, n (%)	HDL (mM)	*P* value	HDL		*P* value
		Median (mean, 5th–95th)		Content ≤ 1.2 n (%)	Content > 1.2 n (%)	
Age (yr)			.302			.111
<54	78 (51.3)	1.115 (1.149, 1.086–1.212)		50 (32.9)	28 (18.4)	
≥54	74 (48.7)	1.185 (1.797, 1.129–1.266)		38 (25.0)	36 (23.7)	
Menarche			.647			.163
<15	86 (56.6)	1.115 (1.163, 1.098–1.228)		54 (35.5)	32 (21.1)	
≥15	66 (43.4)	1.175 (1.185, 1.120–1.251)		34 (22.3)	32 (21.1)	
Marriage age			.111			.307
<22	62 (40.8)	1.105 (1.124, 1.055–1.193)		39 (25.7)	23 (15.1)	
≥22	88 (57.9)	1.165 (1.199, 1.138–1.260)		48 (31.6)	40 (26.3)	
Children			.621			.611
<1	63 (41.4)	1.160 (1.186, 1.123–1.250)		38 (25.0)	25 (16.4)	
>2	89 (58.6)	1.130 (1.163, 1.097–1.229)		50 (32.9)	39 (25.7)	
Histologic type			***.019***			***.016***
Non-serous	47 (30.9)	1.050 (1.029, 0.921–1.125)		34 (13.8)	13 (10.5)	
Serous	105 (69.1)	1.240 (1.287, 1.234–1.340)		54 (44.1)	51 (31.6)	
Difference			.384			.830
Well	18 (11.8)	1.236 (1.259, 1.124–1.400)		9 (5.9)	9 (5.9)	
Moderately	34 (22.4)	1.135 (1.203, 1.091–1.315)		19 (12.5)	15 (9.9)	
Poorly	90 (59.2)	1.145 (1.161 (1.101–1.220)		52 (34.2)	38 (25.0)	
Clinical stage			.848			
I–II	16 (10.5)	1.100 (1.184, 1.037–1.331)		11 (7.2)	5 (303)	.369
III–IV	135 (88.8)	1.150 (1.170, 1.120–1.219)		77 (50.7)	58 (38.1)	

Bold-italic values represent statistically significant (*P* < .05).

HDL = high-density lipoprotein.

### 3.3. Correlations of serum HDL level and progression-free and overall survival rate

**Figure 3.**
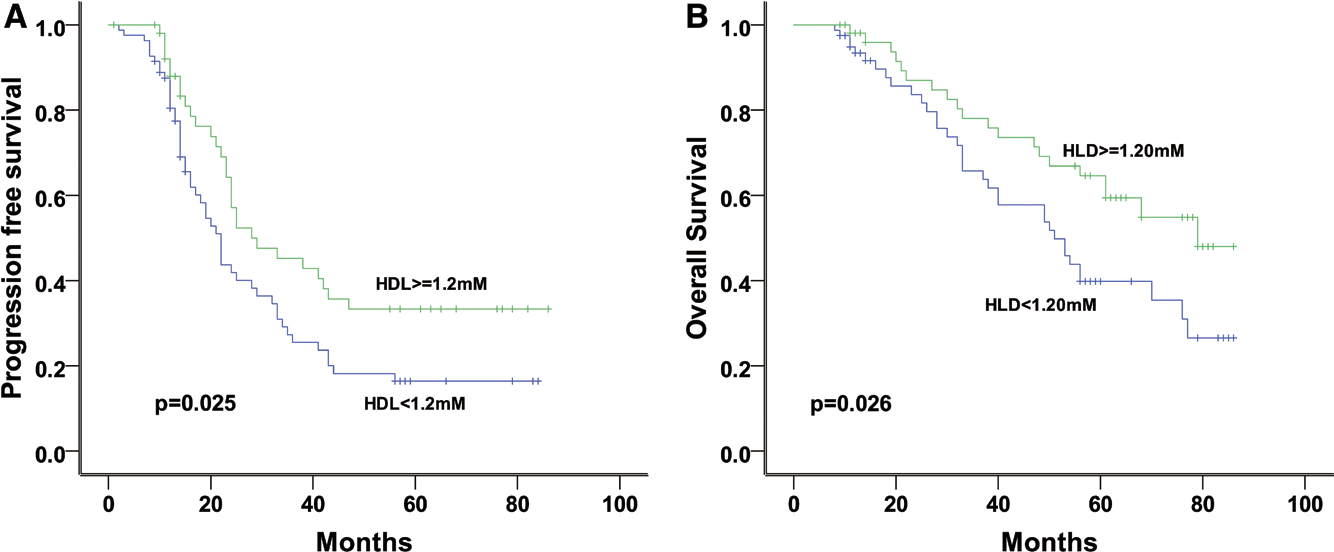
Correlations of serum HDL level with PFS or OS. Graphics show the patients with high serum HDL level had longer PFS (A) and three-year OS (B). *P* < .05 indicates the statistical significance. HDL = high-density lipoprotein, OS = overall survival, PFS = progression-free survival.

Of the 147 patients with follow-up data, 110 experienced disease progression and 88 (80%) died. Kaplan–Meier analysis and log-rank test estimated a 3-year progression-free survival rate of 33.9% and an overall survival rate of 71.3%. The results of the analysis of the association between clinical and pathological factors and prognosis are summarized in Table 2. No significant correlation was observed between the clinical characteristics of age (*P* = .838), age at menarche (*P* = .252), age at marriage (*P* = .116), number of children (*P* = .511), patient pathology, tumor stage (*P* = .126), and progression-free survival. However, we found a significant difference in progression-free survival between patients with stage I to II and stage III to IV disease. Interestingly, our data showed that the median progression-free survival was longer in patients with higher serum HDL levels (28 months) than in patients with lower serum HDL levels (22 months, *P* = .025), and 3-year overall survival was 78.1% for patients with higher HDL levels versus 66.5% for patients with lower serum HDL levels, with medians of 51 and 79 months, respectively (*P* = .026, Fig. 3). Cox proportional hazards regression analysis indicated that the serum HDL level was an independent prognostic factor for patients with ovarian cancer (Table 3). In addition, serum HDL levels were found to be associated with prognosis, that is, longer progression-free survival (hazard ratio, 0.276 [95% CI, 0.110–0.694]) and overall survival (hazard ratio = 0.228 [95% CI = 0.072–0.717], *P* = .025). Increased serum HDL levels were significantly correlated with progression-free and overall survival (both *P* < .05).

**Table 2:** Kaplan–Meier analysis of the correlation between clinic-pathological parameters and survivals for ovarian cancer.

Vaiables	Categories	3-year PFS Rate, %	Median PFS months	Log-rank X2	*P* value	3-year OS Rate, %	Median OS months	Log-rank X2	*P* value
Age (yr)									
<55	78 (51.3)	32.9	24	0.042	.838	79.7	68	1.143	.285
≥55	74 (48.7)	34.9	25			62.9	56		
Menarche									
<15	86 (56.6)	35.3	24	0.252	.616	69.1	53	1.356	.244
≥15	66 (43.4)	31.8	25			74.6	-		
Marriage age									
<22	62 (40.8)	45.2	32	2.465	.116	67.5	56	0.383	.536
≥22	88 (57.9)	27.8	22			75.4	68		
Children									
<1	63 (41.4)	33.1	22	0.431	.511	60.1	76	0.836	.361
>2	89 (58.6)	34.3	25			69.3	56		
Histologic type									
Non-serous		40.8	33	0.216	.642	51.4	38	1.836	.175
Serous		31.5	23			78.4	68		
Difference									
Well	18 (11.8)	51.3		4.146	.126	79.8	50	7.731	***.021***
Moderately	34 (22.4)	34.6	23			65.6	54		
Poorly	90 (59.2)	25.3	25			62.2	44		
Clinical stage									
I–II	16 (10.5)	82.1	-	8.7051	***.003***	84.4	-	4.363	***.037***
III–IV	135 (88.8)	28.5	23			69.4	56		
HDL									
≤1.2	88 (57.9)	25.5	22	4.992	***.025***	66.5	51	4.925	***.026***
>1.2	64 (42.1)	45.2	28			78.1	79		

Bold-italic values represent statistically significant (*P* < .05).

HDL = high-density lipoprotein, OS = overall survival, PFS = progression-free survival.

**Table 3:** Multivariate analysis of potential prognosis value of serum HDL for ovarian cancer.

Variables	Categories	HR	95% CI	*P* value
Progression-free survival				
Age	≥55 vs<55	1.524	0.730–3.182	.262
Menarche	≥15 vs <15	0.692	0.422–1.135	.524
Marriage age	≥22 vs <22	1.532	0.875–2.681	.135
Menopause	No vs Yes	2.438	1.140–5.214	.22
Difference	Well vs Moderately vs Poorly	0.892	0.628–1.267	.524
Clinical stage	I–II vs III–IV	3.550	1.057–11.928	***.040***
Histologic type	Non-serous vs serous	0.992	0.804–1.223	.937
Children	>2 vs <1	1.044	0.603–1.805	.879
HDL	>1.2 vs **≤**1.2	0.276	0.110–0.694	***.006***
Overall survival				
Age	≥55 vs<55	1.322	0.502–3.477	.572
Menarche	≥15 vs <15	0.632	0.345–1.157	.137
Marriage age	≥22 vs <22	0.871	0.448–1.696	.685
Menopause	No vs Yes	1.687	0.626–4.551	.301
Difference	Well vs Moderately vs Poorly	0.626	0.417–0.940	***.024***
Clinical stage	I–II vs III–IV	5.925	1.368–25.658	***.017***
Histologic type	Non-serous vs serous	1.203	0.934–1.550	.151
Children	>2 vs <1	1.359	0.633–2.918	.432
HDL	>1.2 vs **≤**1.2	0.228	0.072–0.717	***.011***

Bold-italic values represent statistically significant (*P* < .05).

HDL = high-density lipoprotein, HR = hazard ratio.

## 4. Discussion

Epidemiological studies have shown that the occurrence and development of many malignant tumors are associated with obesity, and the increased cell proliferation that occurs during the pathogenesis of malignant tumors can lead to abnormally elevated lipid metabolism and changes in lipoprotein levels. High lipid metabolism in cancer cells may also increase circulating total cholesterol and low-density lipoprotein levels.

Interestingly, HDL eliminates excess total cholesterol from the circulation by reverse transport to the liver. HDL may also be involved in tumor formation via the mitogen-activated protein kinase pathway, thus regulating cell cycle, apoptosis, cytokine production, and antioxidants.^[15–17]^ In addition to alterations in the expression of oncogenes and tumor suppressor genes, inflammation and oxidative stress also affect cellular proliferation and malignant transformation during ovarian cancer development.^[18,19]^ In these settings, HDL may be a target candidate that is capable of inhibiting tumor growth by modulating innate immune activity during tumorigenesis or by upregulating antioxidant activity to inhibit cell proliferation during tumor formation.^[20]^ In addition, HDL and its polypeptides also play important roles in regulating tumor cell proliferation, differentiation, motility, and invasion by inhibiting the activation of vascular endothelial growth factor and basic fibroblast growth factor, as well as by regulating the Akt and ERK1/2 signaling pathways.^[21]^ Proteins that control HDL and its derivatives are important for the regulation of tumor progression.^[22]^ Indeed, negative correlations between HDL and the incidence of rectal cancer have been reported,^[23]^ and high serum HDL levels have been reported to be associated with reduced cancer risk.^[24,25]^

On the other hand, studies have found that increased expression or activity of apolipoprotein A correlates with reduced tumor burden and improved overall survival and prognosis of cancers, including ovarian cancer.^[26,27]^ apolipoprotein A is the main component of HDL, which functions to maintain HDL structural stability, promote cholesterol efflux from macrophages, stimulate the reverse transport of lipids, inhibit low-density lipoprotein oxidation, and promote the removal of toxic phospholipids.^[28]^ This evidence suggests the potential role of HDL as a biomarker or therapeutic target for ovarian cancer. Notably, a case-control study reported that serum HDL was lower in patients with newly diagnosed lung cancer than in healthy controls, suggesting that low HDL is associated with cancer occurrence.^[29]^ Another prospective study reported dyslipidemia in the majority of 15,792 participants and lung cancer in 259 patients with low HDL levels during 13 years of follow-up, indicating that low HDL levels were associated with an increased incidence of lung cancer.^[8]^ However, it is still unclear whether reduced HDL levels increase the incidence of lung cancer or whether lung cancer causes a decrease in HDL levels.

Nevertheless, our study showed that serum HDL level was significantly lower in ovarian cancer patients than in patients with benign ovarian tumors, and the ROC curve analysis demonstrated an area under the curve of 0.735, indicating a diagnostic value of serum HDL level that can distinguish benign from malignant ovarian cancer patients. Our data further showed that serum HDL levels correlate with pathological types of ovarian cancer, but not with age, age at menarche or marriage age, number of children, tumor grade, or clinical stage. Patients with higher serum HDL levels had better progression-free and overall survival than those with lower HDL levels. These results indicate the potential of serum HDL levels as an independent prognostic factor for ovarian cancer and that the increase in HDL levels may reduce the risk of disease recurrence.

## 5. Conclusion

Our data suggest that serum HDL level may serve as a useful indicator for the diagnosis and prognosis of ovarian cancer.

## Acknowledgements

This study was supported by Zhejiang Provincial Medicine and Health Science Fund (Nos. 2018KY316 and 2021KY438).

## Author contributions

**Conceptualization:** Jiang Hongyan, Chen Pengcheng, Feng Jianguo.

**Data curation:** Jiang Hongyan.

**Formal analysis:** Jiang Hongyan.

**Funding acquisition:** Chen Pengcheng, Feng Jianguo.

**Investigation:** Zhu Chihong, Qian Xiaoqian.

**Methodology:** Zhu Chihong, Feng Jianguo.

**Resources:** Qian Xiaoqian.

**Software:** Wan Danying.

**Supervision:** Zhu Chihong, Wan Danying.

**Validation:** Chen Pengcheng, Zhu Chihong, Wan Danying.

**Writing – original draft:** Jiang Hongyan, Chen Pengcheng.

**Writing – review & editing:** Feng Jianguo.
